# Evolution of therapy for autoimmune diseases in pregnancy: a retrospective study from 2000 to 2023

**DOI:** 10.3389/fmed.2026.1716385

**Published:** 2026-03-18

**Authors:** Cristina Garufi, Francesca Rizzo, Viviana Matys, Ester Garufi, Angela Botta, Valerio Andreozzi, Silvia Salvi, Tullio Ghi, Sara De Carolis

**Affiliations:** 1 Department of Internal Clinical Sciences, Anesthesiology and Cardiovascular Sciences, Sapienza University of Rome, Rome, Italy; 2 Department of Women and Child Health, Women Health Area, Fondazione Policlinico Universitario Agostino Gemelli, IRCCS, Rome, Italy; 3 Department of Maternal, Infantile and Urological Sciences, Sapienza University of Rome, Rome, Italy; 4 Department of Anesthesiology and Intensive Care Medicine, Catholic University of the Sacred Heart, Rome, Italy; 5 Department of Orthopaedics, Sapienza University of Rome, Rome, Italy

**Keywords:** autoimmune diseases, corticosteroids, hydroxychloroquine, low-doseaspirin, low-molecular-weight heparin, pregnancy

## Abstract

**Introduction:**

Treatment options for rheumatic diseases in pregnancy have consistently changed over the years. The most common therapies for autoimmune diseases during pregnancy include low-dose aspirin (LDA), low-molecular-weight heparin (LMWH), steroids, and hydroxychloroquine (HCQ). Therapy choice is based on obstetrical history, presence of antiphospholipid antibodies, and disease severity and refractoriness. Over the years, we have observed a decrease in glucocorticoid administration and an increase in HCQ administration. Glucocorticoid therapy during pregnancy can increase the risk of premature rupture of membranes and intrauterine growth restriction. In mothers, it is correlated with gestational hypertension, diabetes, osteoporosis, and infections. HCQ appears safe in pregnancy for fetal development and growth; it may improve pregnancy outcomes and reduce the risk of preeclampsia and autoimmune disease flares.

**Methods:**

We conducted a retrospective study of 266 pregnant women affected by autoimmune diseases (systemic lupus erythematosus, antiphospholipid syndrome, Sjogren’s Syndrome, connective tissue disease) referred to our center from 2000 to 2023.

**Results:**

We observed substantial stability in LDA use, ranging from 100% in 2000–2003 to 96% in 2020–2023 (mean 88%; p 0.54). Regarding LMWH, we observed a modest but consistent increase, starting from 25% in 2000–2003 to 61% in 2020–2023 (mean 50%; *p* = 0.18). A greater increase was observed in HCQ use, which passed from 13% in 2000–2003 to 40% in 2020–2023 (mean 30%; *p* 0.62). Lastly, we observed a statistically significant decrease in steroid use, from 50% in 2000–2003 to 13% in 2020–2023 (mean 33%; *p* < 0.01).

**Conclusion:**

These changes in therapy reflect increased knowledge in autoimmune diseases during pregnancy, with a more consistent number of women able to have and carry a pregnancy despite their disease severity. Over the years, our center had offered the best therapeutic management to achieve a good obstetrical outcome and minimize the incidence of obstetrical complications.

## 1 Introduction

The management of autoimmune rheumatic diseases during pregnancy aims to achieve two primary objectives: firstly, to prevent the reactivation of the underlying autoimmune disease, and secondly, to improve the obstetrical outcome while minimizing the risk of pregnancy complications.

Over the years, treatment options for rheumatic diseases in pregnant women have evolved significantly. In addition to immunosuppressive drugs allowed, the most common treatment for autoimmune diseases in pregnancy includes these important drugs: low-dose aspirin (LDA) and low molecular weight heparin (LMWH), often in association with steroids and/or hydroxychloroquine (HCQ). The choice of therapy is guided by an individual obstetrical history - such as previous history of preeclampsia, miscarriages, or intra-uterine fetal demise (IUFD) - as well as the presence of antiphospholipid antibodies, the severity and refractoriness of the rheumatic condition.

Low-dose aspirin is administered once daily, after dinner, at a dose ranging from 82 to 160 mg (1). Subcutaneous administration of LMWH is performed at two distinct dosing regimens: a prophylactic dose of 4,000 IU once daily and a weight-adjusted therapeutic dose administered twice daily. Therapeutic dosing is reserved for specific clinical conditions, such as active thrombosis, the presence of other thrombophilias, or antiphospholipid syndrome (APS) with a history of thrombosis (1, 2). Prednisone, prednisolone, and methylprednisolone are commonly used short-acting glucocorticoids, while dexamethasone and betamethasone are commonly used long-acting agents. Dexamethasone and betamethasone reach higher fetal concentrations than prednisone and prednisolone (3).

The typical daily dosage of HCQ for rheumatic diseases ranges from 200 to 400 mg. To mitigate the risk of retinal toxicity, HCQ dosing is adjusted based on actual body weight, with a maximum daily dose of 5 mg/kg/day, not exceeding 400 mg (4). HCQ, initially used against malaria, has been widely adopted for treating systemic autoimmune diseases (5). It exerts its effects by inhibiting B-cell activation, suppressing complement activation, and disrupting endolysosomal functions (6). These actions collectively reduce inflammation (7). In APS, HCQ demonstrates multiple beneficial effects. It reduces antiphospholipid antibody (aPL) binding to the placenta, modulates endothelial cell activation, and potentially decreases antibody production (8, 9).

An analysis that examines the trends in therapy across a heterogeneous population of systemic rheumatic diseases provides a valuable perspective for this study. Many autoimmune rheumatic diseases share a common etiology and frequently exhibit overlapping symptoms and laboratory findings. Additionally, systemic autoimmune disorders do not always present as distinct autoimmune syndromes; in fact, approximately 25% of patients may exhibit undifferentiated systemic rheumatic diseases and/or overlap syndromes, making definitive diagnoses challenging (10, 11).

Attention toward therapies for pregnant women with systemic autoimmune diseases has significantly increased in recent years. Moreover, the awareness of the heightened obstetric risks faced by these patients is growing. These diseases can negatively impact fertility, fecundity, and pregnancy outcomes, including increased risk of disease flares and both maternal and fetal complications (12, 13).

The present study aimed to analyze the evolution of therapy for autoimmune diseases in pregnancy in our center.

## 2 Materials and methods

In this retrospective study, we analyzed data from 269 singleton pregnancies of patients diagnosed with systemic autoimmune diseases who were referred to our center (Fondazione Policlinico Gemelli IRCCS) from 2000 to 2023.

Our study cohort included a heterogeneous group of autoimmune diseases. Antiphospholipid syndrome was diagnosed according to Sapporo 2006 and Sydney 2004 criteria (14). This cohort also included a group of patients with persistent antiphospholipid antibodies and “non-classical” criteria for APS diagnosis (15). The diagnosis of systemic lupus erythematosus (SLE) was made according to the 2019 EULAR/ACR classification criteria (16). Sjögren Syndrome was diagnosed based on the 2016 ACR/EULAR classification criteria for Sjögren’s syndrome (17). The diagnosis of connective tissue disease (CTD) should be suspected in a patient presenting with overlapping features of different systemic rheumatic diseases (18).

For our analysis of the data, only pregnancies that resulted in live births were included; therefore, two patients with pregnancy loss were excluded. These two patients were diagnosed with systemic lupus erythematosus (SLE). One patient experienced intrauterine fetal demise (IUFD) at 27 weeks’ gestation while receiving low-dose aspirin (LDA) and low-molecular-weight heparin (LMWH). The other patient experienced a late miscarriage during treatment with LDA, LMWH, and hydroxychloroquine (HCQ).

One of the limitations of our study was the inability to determine the rate of early miscarriage, as some patients with early-stage miscarriages did not come under our observation and, therefore, do not appear in this case history or were lost.

We evaluated the temporal trends relating to the use of each therapeutic regimen (LDA, LMWH, HCQ, and steroids) over the study period.

Our primary objective was to evaluate the obstetrical outcome associated with the different therapeutic regimens, including LDA, LMWH, HCQ, and steroids. We specifically investigated several parameters related to delivery outcome, including gestational age at delivery (preterm deliveries were defined as <37 weeks). Additionally, we assessed birth weight, focusing on infants weighing less than 2,500 g and those falling below the 10th percentile for birth weight as per an established Italian population-based study (19).

Furthermore, we estimated the frequency of pregnancy-related complications for each treatment group, in terms of gestational diabetes, gestational hypertension, and preeclampsia.

To understand the risk factors associated with the severity of autoimmune diseases and their impact on pregnancy complications, we assessed several variables for each therapy group. These included: maternal age at birth (specifically, ages ≤ 34 years, 35–39 years and ≥40 years), race group, presence of overlap syndromes (where patients presented with more than one autoimmune disease), the presence of triple aPL positivity, antinuclear antibodies (ANA) positivity, extractable nuclear antigens antibodies (ENA) positivity, history of previous miscarriages (defined as ≥2), history of previous IUFD, alterations in the pulsatility index of the uterine artery, serum levels of complement proteins (to establish low complementemia), chronic hypertension, diabetes mellitus, use of steroid therapy, and treatment lacking LDA.

To enhance the understanding and management of pregnancies complicated by autoimmune diseases, we corrected the obstetrical outcome for each treatment category based on the presence of the aforementioned risk factors, allowing for a nuanced understanding of their influence on pregnancy outcomes in our patient population.

A logistic regression analysis was performed calculating the odds ratios for primary and secondary endpoints, adjusted for the following risk factors: maternal age at delivery, race, presence of overlap syndrome, history of more than two miscarriages, history of IUFD, presence of triple aPL positivity, ANA positivity, ENA positivity, low serum complement levels (C3, C4), corticosteroid use and treatment lacking LDA. Adjustment for the following variables, also considered as risk factors, was not possible due to the small number of positive cases or high number of missing data in the dataset: abnormal uterine artery pulsatility, chronic hypertension, pre-gestational diabetes mellitus. Another limitation of this analysis is that odds ratios could not be calculated for some comparisons due to the absence of cases with birth weights below 2,500 g in the non-LDA group. We also had to exclude the outcome “weight below the 10th percentile” and “preeclampsia” due to the too small number of positive cases.

Continuous data results were presented as mean ± SD, and categorical data were expressed as absolute values and percentages. Continuous variables with normal distribution were analyzed by applying the *T*-Test, while nominal variables were analyzed by cross-tabulation and Chi-square with Yates correction. Generalized logistic regression was used to adjust for confounding factors in the analysis of pregnancy outcomes. Values of *p* < 0.05 were considered significant. IBM SPSS, Jamovi statistical software and Microsoft Office Excel were employed for analyses.

## 3 Results

A total of 269 patients were included in the study. The mean age was 34.6 ± 4.8 years, and 50.2% of patients were aged ≥ 35 years. Baseline demographic, clinical, and immunological characteristics are summarized in Table 1.

**TABLE 1:** Baseline demographic, clinical and immunological characteristics of the study population.

Variable	Overall (*N* = 269)
Age, mean ± SD (years)	34.6 ± 4.8
Age ≥ 35 years, *n* (%)	135 (50.2)
Primary antiphospholipid syndrome (pAPS), *n* (%)	122 (45)
Systemic lupus erythematosus (SLE), *n* (%)	85 (32)
SLE associated with secondary antiphospholipid syndrome (sAPS), *n* (%)	30 (11)
Sjögren’s disease (SD), *n* (%)	34 (12)
SD associated with secondary antiphospholipid syndrome (sAPS), *n* (%)	9 (3)
SD associated with SLE, *n* (%)	2 (0.7)
Connective tissue diseases (CTD), *n* (%)	31 (12)
CTD associated with secondary antiphospholipid syndrome (sAPS), *n* (%)	1 (0.3)
Anticardiolipin antibodies (aCL) positive, *n* (%)	80 (29.7)
Lupus anticoagulant (LAC) positive, *n* (%)	94 (34.9)
Anti-β2 glycoprotein I antibodies positive, *n* (%)	60 (22.3)
Triple antibody positivity, *n* (%)	28 (10.4)
≥2 previous miscarriages, *n* (%)	41 (15.2)
Diabetes mellitus, *n* (%)	6 (2.2)
Chronic hypertension, *n* (%)	2 (0.7)

**FIGURE 1:**
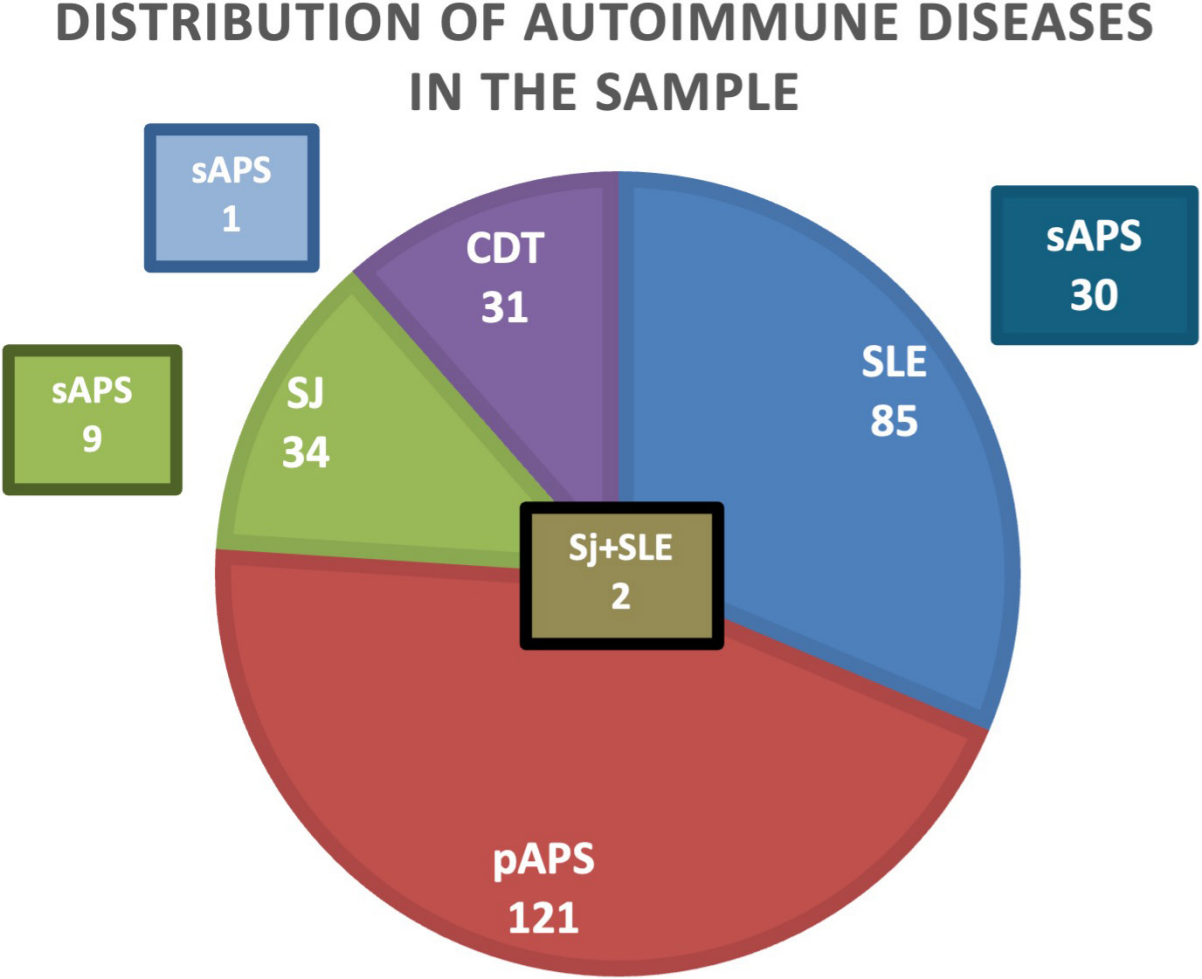
Distribution of autoimmune diseases in the sample. SLE, systemic lupus erythematosus; pAPS, primary antiphospholipid syndrome; sAPS, secondary Antiphospholipid syndrome; SJ, Sjogren Syndrome; CTD, connective tissue disease.

Disease’ distribution was as follows: 85 women were affected by systemic lupus erythematosus (SLE) (32%); 122 women were affected by primary antiphospholipid syndrome (pAPS) (45%) - including 32 patients with aPL; 34 women were affected by Sjogren Syndrome (SJ) (12%); 31 women were affected by connective tissue disease (CTD) (12%). Some patients had overlap syndromes, in fact: 30 of 83 patients with SLE had secondary APS (sAPS); 9 of 30 patients affected by SJ had sAPS; 2 women had both SJ and LES; 1 woman had sAPS and CTD (Figure 1).

Primary antiphospholipid syndrome (pAPS) was diagnosed in 123 patients (45.7%), while 146 patients (54.3%) had secondary APS (sAPS). Among sAPS patients, the underlying autoimmune disease was systemic lupus erythematosus in 84 cases, Sjögren’s syndrome in 30 cases, and other connective tissue diseases in 32 cases. Overall, 44 patients (16.0%) had an additional autoimmune disease.

**FIGURE 2:**
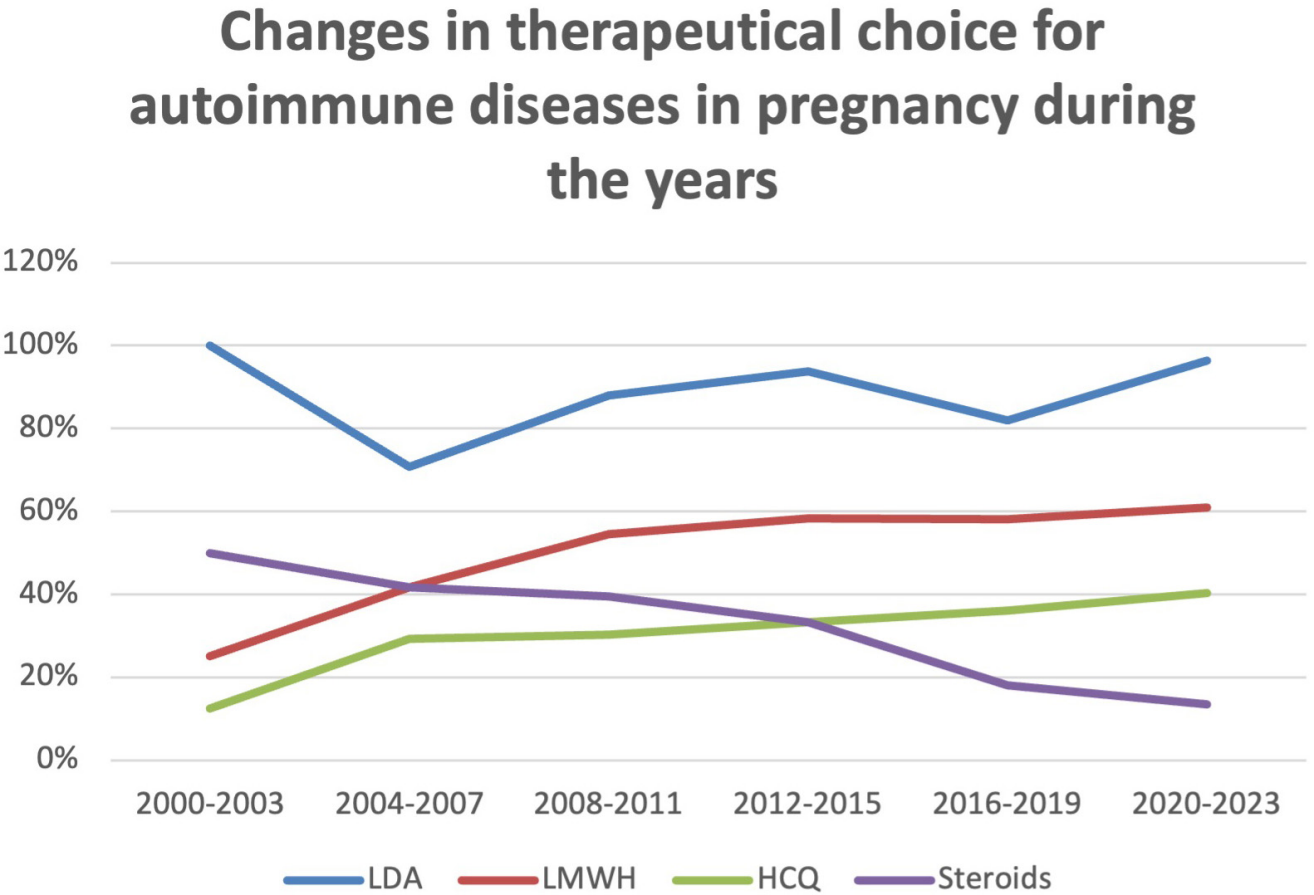
Changes in therapeutical choice for autoimmune diseases in pregnancy during the years; percentage of use of each drug. LDA, low dose aspirin; LMWH, low molecular weight heparin; HCQ, hydroxychloroquine.

Concerning the evolution of therapy over the years, we observed a substantial stability in the use of LDA, ranging from 100% in 2000–2003 period to 96% in 2020–2023 period (mean 88% ± 11%; *p*-value = 0.54). Regarding LMWH, we observed a modest but constant increase, starting from 25% of use in 2000–2003 period to 61% in 2020–2023 period (mean 50% ± 14%; *p*-value = 0.18). A larger increase was observed in the use of HCQ, which passed from 13% in 2000–2003 period to 40% in 2020–2023 period (mean 30% ± 10%; *p*-value = 0.62). Lastly, we observed a statistically significant decrease in the use of steroids, from 50% in 2000–2003 period to 13% in 2020–2023 period (mean 33% ± 14%; *p* < 0.01) (Figure 2 and Table 2).

**TABLE 2:** Changes in the choice for treatment of autoimmune diseases in pregnancy during the years.

Years (from–to)	LDA	LMWH	HCQ	Steroids	Number of cases
2000–2003	100%	25%	13%	50%	7
2004–2007	71%	42%	29%	42%	22
2008–2011	88%	55%	30%	39%	31
2012–2015	94%	58%	33%	33%	42
2016–2019	82%	58%	36%	18%	89
2020–2023	96%	61%	40%	13%	78
Mean	88%	50%	30%	33%	
SD	11%	14%	10%	14%	
*P*	0.54	0.18	0.62	0.01	

LDA, low dose aspirin; LMWH, low molecular weight heparin; HCQ, hydroxychloroquine.

Regarding the primary obstetric outcome, the analysis demonstrated no statistically significant differences in gestational age at delivery, birth weight, or birth weight percentile across the treatment groups receiving LDA, HCQ, and LMWH. In contrast, a statistically significant difference was observed in the steroid treatment group for deliveries occurring before 37 weeks of gestation (19% vs. 10%, *p* 0.05) (Table 3).

**TABLE 3:** Primary outcome.

Study population	LDA		LMWH		Steroids		HCQ	
	Yes	No	Yes	No	Yes	No	Yes	No
Number of cases	250	17	161	106	63	204	102	165
Week at delivery < 37	31	2	22	11	12	21	15	18
%	12%	12%	14%	10%	19%	10%	15%	11%
*P*-value	0.99		0.44		0.05		0.34	
Birth weight < 2,500 g	36	1	24	13	13	24	16	21
%	14%	6%	15%	12%	21%	12%	16%	13%
*P*-value	0.32		0.54		0.08		0.49	
Birth weight percentile < 10	20	0	12	5	4	13	6	14
%	8%	0%	7%	5%	6%	6%	6%	8%
*P*-value	0.24		0.37		0.99		0.46	

LDA, low dose aspirin; LMWH, low molecular weight heparin; HCQ, hydroxychloroquine.

Concerning our secondary endpoints, such as gestational diabetes, gestational hypertension and preeclampsia, no significant differences in the rates of these complications were identified among the four different treatments (LDA, LMWH, steroids, and HCQ). In patients treated with steroids, a moderate increase in the risk of gestational diabetes (19% vs. 13%; *p*-value = 0.22), gestational hypertension (21% vs. 15%; *p*-value = 0.31), and preeclampsia (8% vs. 3%; *p*-value = 0.13) was observed. However, none of these differences reached statistical significance. In case of HCQ therapy, we observed a very slight improvement in terms of the incidence of gestational diabetes (13% vs. 15%; *p*-value = 0.57), gestational hypertension (14% vs. 18%; *p*-value = 0.34), and preeclampsia (4% vs. 5%; *p*-value = 0.72), but none of these differences reached statistical significance (Table 4).

**TABLE 4:** Secondary outcome: obstetrical complications.

Study population	LDA		LMWH		Steroids		HCQ	
	Yes	No	Yes	No	Yes	No	Yes	No
Number of cases	250	17	161	106	63	204	102	165
Gestational diabetes	36	2	20	18	12	26	13	25
%	14%	12%	12%	17%	19%	13%	13%	15%
*P*-value	0.76		0.29		0.22		0.57	
Gestational hypertension	43	1	31	13	13	31	14	30
%	17%	6%	19%	12%	21%	15%	14%	18%
*P*-value	0.23		0.13		0.31		0.34	
Preeclampsia	12	0	10	2	5	7	4	8
%	5%	0%	6%	2%	8%	3%	4%	5%
*P*-value	0.35		0.10		0.13		0.72	

LDA, low dose aspirin; LMWH, low molecular weight heparin; HCQ, hydroxychloroquine.

**FIGURE 3:**
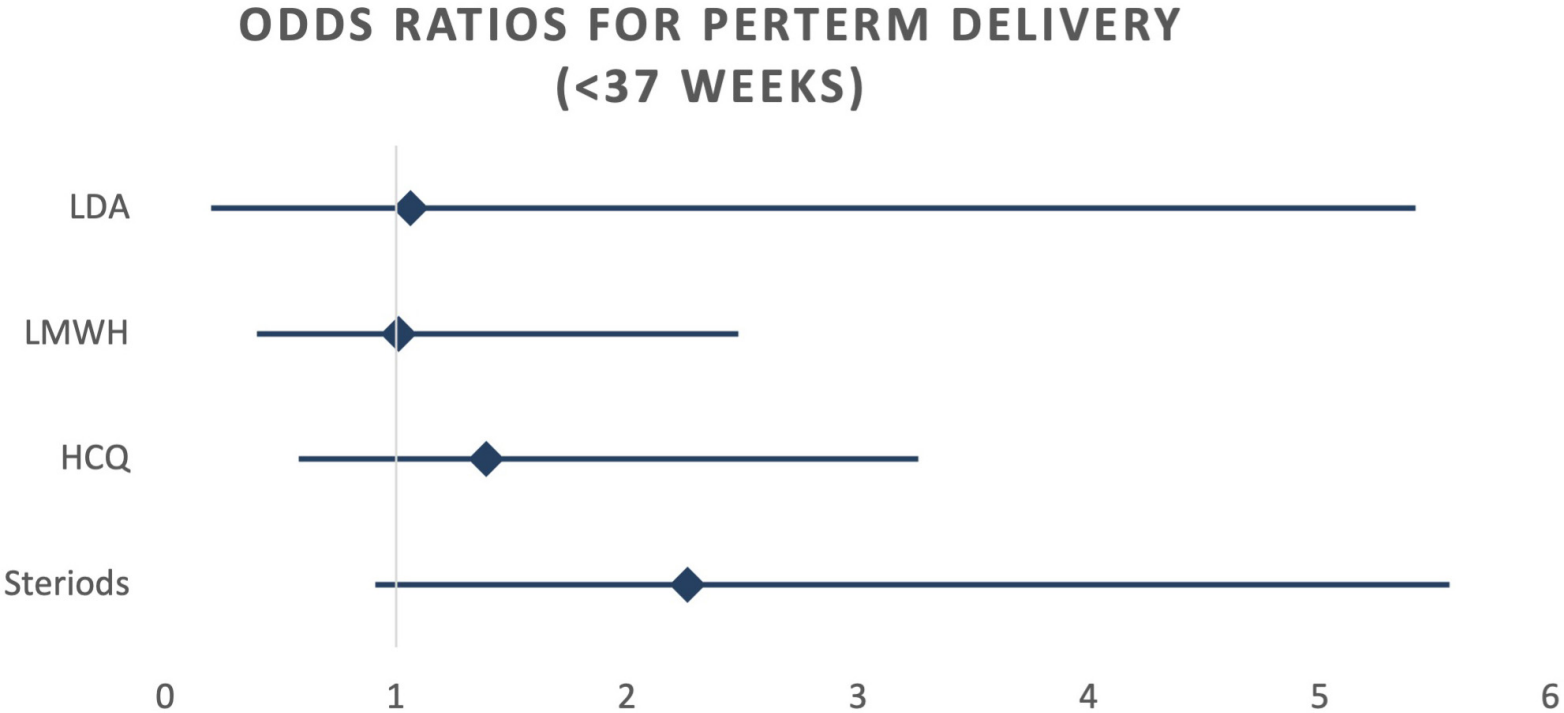
Risk adjusted odds ratios for preterm delivery (<37 weeks). LDA, low dose aspirin; LMWH, low molecular weight heparin; HCQ, hydroxychloroquine.

**FIGURE 4:**
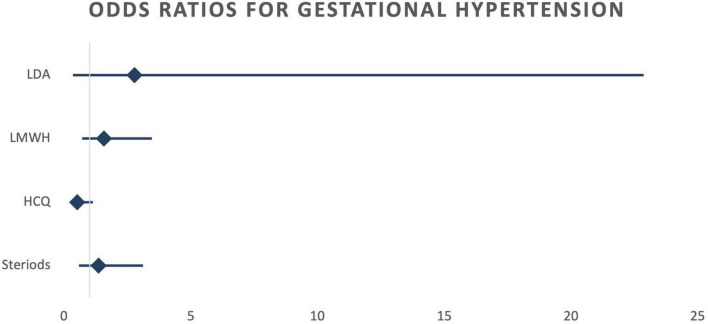
Risk adjusted odds ratios Gestational hypertension. LDA, low dose aspirin; LMWH, low molecular weight heparin; HCQ, hydroxychloroquine.

When calculating odds ratios, no statistically significant differences were detected among the four treatment groups, regardless of whether crude or risk-adjusted odds ratios were considered (Table 4). However, a notable trend emerged: there was an increased occurrence of preterm birth (<37 weeks) in the steroid therapy group. While not statistically significant, the 95% confidence interval approached the significance threshold (OR 2.09; CI 0.96–4.54). This trend persisted even after adjusting for potential confounding factors using logistic regression (OR 2.26; CI 0.92–5.55) (Figure 3). Conversely, a reduction in the occurrence of gestational hypertension was observed in the HCQ group (OR 0.71; 95% CI 0.36–1.42). This reduction became more pronounced after adjusting for risk factors (OR 0.52; 95% CI 0.24–1.13), although it did not reach statistical significance in either case (Figure 4).

## 4 Discussion

Over the years, we observed a substantial stability in the use of LDA, with a good adherence to this prophylaxis. Further, we noticed a slight increment in the use of LMWH, a consistent decrease in glucocorticoids administration, and an increase in HCQ administration.

It is well-known that high-dose aspirin is used as an analgesic and anti-inflammatory agent in the management of rheumatic disease outside of pregnancy, but its use is not allowed during pregnancy. On the contrary, low-dose aspirin should be started preconceptionally, as part of the management of antiphospholipid syndrome and in all systemic autoimmune diseases for the prevention of preeclampsia and intrauterine growth restriction. This recommendation is supported by major guidelines globally, including those from the American College of Obstetricians and Gynecologists, the World Health Organization, the National Institute for Health and Care Excellence, and the European Alliance of Associations for Rheumatology (EULAR) (1, 20–22).

In patients with antiphospholipid syndrome, the initiation of LMWH should occur promptly upon confirmation of pregnancy (1). Its use is also advocated at prophylactic doses in individuals with SLE who have a history of thrombosis, those with obstetric criteria for antiphospholipid syndrome (23). A recent retrospective study conducted by Tan et al. found that the application of LMWH serves as an independent protective factor for fetal survival, also in cases of primary Sjögren’s syndrome, further endorsing its integration into treatment protocols for this condition (24). Finally, it should be considered that many patients with systemic autoimmune diseases have an obstetric indication for prophylaxis with LMWH, such as history of intrauterine fetal demise, history of early and severe preeclampsia, history of early intrauterine growth restriction (12). The scientific literature on this subject remains inconclusive, although some recent studies have suggested a potential benefit of heparin in these settings (25–27).

Regarding the use of steroids in pregnancy, its use showed a statistically significant decrease during the years in our study group. Some evidence suggested that glucocorticoid administration during gestation could be associated with a heightened risk of premature rupture of the membranes (28) and intrauterine growth restriction (29). Furthermore, maternal exposure to glucocorticoids has been correlated with several pregnancy-related complications, including gestational hypertension, gestational diabetes mellitus, osteoporosis, and increased susceptibility to infection (30). Focusing on the association between preterm birth and glucocorticoid treatment, this compelling recent investigation by Shimada et al. (31) investigated 74 pregnancies complicated by SLE. A dose-dependent relationship between glucocorticoid administration and preterm delivery was observed, suggesting that autoimmune disease should be managed with the minimal effective dose of steroids (31). Conversely, a 2017 systematic review found limited evidence to support a causal relationship between systemic corticosteroid use during pregnancy and an increased risk of preterm birth, low birth weight, or preeclampsia. This limitation arises from the significant heterogeneity observed in the available data and the uncertainty surrounding the extent to which the underlying maternal disease contributes to these potential adverse outcomes (32).

In addition to the well-known adverse effects of glucocorticoids in the general population, long-term use is consistently associated with a dose-dependent and time-dependent increase in the risk of developing additional comorbidities that can reduce life expectancy in chronically treated patients. Notably, there is a well-established association with the onset of diabetes mellitus in previously non-diabetic individuals, premature atherosclerosis, and potential dyslipidemia (33–35).

The administration of antenatal steroids (ACS) is a well-established intervention for the prevention of respiratory distress syndrome in preterm infants, also in pregnancies of patients without autoimmune diseases. However, while beneficial in cases of imminent preterm birth, glucocorticoid therapy is associated with a range of potential adverse effects. Animal studies suggest that ACS may promote premature organ differentiation at the expense of cell proliferation, potentially predisposing preterm infants to long-term health issues such as diabetes, hypertension, heart failure, and psychiatric disorders (36, 37). Furthermore, late-gestation exposure to supraphysiological corticosteroid levels may disrupt fetal brain development, potentially impacting neurodevelopmental outcomes (38).

On the other hand, HCQ is confirmed to be safe in pregnancy for fetal development and growth (39) and may improve the pregnancy outcomes. Regarding the use of HCQ in pregnancies complicated by APS, our group published one of the first studies proposing its beneficial effect in refractory APS in 2015 (8). Subsequent studies confirmed the utility of HCQ in refractory APS cases, that is, when standard therapy with LDA and LMWH failed to improve obstetric outcomes (40–42). In 2023, a systematic review by Hooper et al. concluded that HCQ, when added to aspirin and heparin, may significantly reduce the risk of antiphospholipid antibody-mediated obstetrical complications in patients with refractory APS (43).

Hydroxychloroquine is widely recommended as a disease-modifying anti-rheumatic drug (DMARD) for SLE patients, helping to prevent flares and improve long-term outcomes (44). The literature supports the use of HCQ in pregnancies complicated by SLE, with several studies demonstrating its potential therapeutic benefits (45–47). According to this 2023 meta-analysis by Hu et al., the addition of HCQ to standard care may potentially reduce the risk of lupus flares and preeclampsia in pregnant women with SLE (48).

Hydroxychloroquine has demonstrated a protective effect in children born to mothers with autoimmune diseases. This therapy reduced the risk and delayed the onset of cutaneous neonatal lupus (49). Furthermore, a growing body of evidence suggests that HCQ may play a role in reducing the risk of fetal/neonatal heart block in pregnancies of patients with anti-SSA/Ro antibodies (50). Thus, its use is recommended in women with positive anti-SSA/Ro antibodies at risk for congenital heart block (50).

Several randomized controlled trials are underway to demonstrate the efficacy of HCQ in reducing the risk of preeclampsia and for the treatment of women with recurrent pregnancy loss, regardless of autoimmune status (6, 51, 52). Current literature suggests that the results of this drug in this setting are promising and may represent an effective element in the management of these pregnancy complications (53).

## 5 Conclusion

Glucocorticoid therapy during pregnancy resulted to be associated with a higher risk of preterm delivery, confirming the preliminary data of the literature. Further, during the years we observed a statistically significant decrease in the use of steroids.

On the contrary, we detected an increase in the use of HCQ, which passed from 13% in the 2000–2003 period to 40% in the 2020–2023 period (mean 30% ± 10%; *p* = 0.62).

While HCQ therapy may offer potential benefits for improving overall obstetrical outcomes, in our study, no significant differences were observed in the analyzed features, as gestational age at delivery, birth weight, birth weight percentiles or incidence of preeclampsia, between groups receiving HCQ or not receiving HCQ. In our opinion, HCQ therapy was reserved for more severe autoimmune diseases, as triple aPL positivity or refractory APS cases, which are associated with worse obstetrical outcomes. Probably, the similar pregnancy outcome in the two groups is just a good result, which can be explained by the beneficial effect of HCQ in those cases having a higher obstetrical risk.

Methodological limitations of our study include a heterogeneous sample, a retrospective study design, and a relatively small sample size. Additionally, the delayed referral of patients during pregnancy precluded the accurate calculation of pregnancy loss rates.

The change in the therapeutic choices is indicative of the ongoing knowledge and heightened clinical interest in the field of autoimmune diseases and pregnancy. Moreover, we highlighted a substantial and consistent increase over the years in the number of women of reproductive age with autoimmune disorders who had pregnancies, irrespective of disease severity. Our center consistently provided tailored therapeutic strategies, with the primary goals of achieving optimal obstetric outcomes, minimizing the occurrence of pregnancy-related adverse events, and preserving maternal health.

Future research with a broader, multicentric and prospective approach needs to generate more robust, long-term data.

## Data Availability

The datasets presented in this article are not readily available because data sharing is not applicable. Requests to access the datasets should be directed to viviana.matys@gmail.com.

## References

[1] Tektonidou MG Andreoli L Limper M Amoura Z Cervera R Costedoat-Chalumeau N et al. EULAR recommendations for the management of antiphospholipid syndrome in adults. *Ann Rheum Dis*. (2019) 78:1296–304. 10.1136/annrheumdis-2019-215213 31092409 PMC11034817

[2] Sammaritano LR Bermas BL Chakravarty EE Chambers C Clowse MEB Lockshin MD et al. 2020 American College of Rheumatology Guideline for the Management of Reproductive Health in Rheumatic and Musculoskeletal Diseases. *Arthritis Care Res*. (2020) 72:461–88. 10.1002/acr.24130 32090466

[3] Beitins IZ Bayard F Ances IG Kowarski A Migeon CJ. The transplacental passage of prednisone and prednisolone in pregnancy near term. *J Pediatr*. (1972) 81:936–45. 10.1016/s0022-3476(72)80547-x 5086721

[4] Marmor MF Kellner U Lai TY Melles RB Mieler WF. American Academy of ophthalmology. recommendations on screening for chloroquine and hydroxychloroquine retinopathy (2016 Revision). *Ophthalmology*. (2016) 123:1386–94. 10.1016/j.ophtha.2016.01.058 26992838

[5] Nirk EL Reggiori F Mauthe M. Hydroxychloroquine in rheumatic autoimmune disorders and beyond. *EMBO Mol Med*. (2020) 12:e12476. 10.15252/emmm.202012476 32715647 PMC7411564

[6] Mekinian A Vicaut E Cohen J Bornes M Kayem G Fain O. Évaluation du bénéfice de l’utilisation d’hydroxychloroquine pour l’obtention d’une grossesse à terme non compliquée en cas de syndrome des antiphospholipides primaire : étude de phase II multicentrique randomisée en double insu versus placebo, HYDROSAPL [Hydroxychloroquine to obtain pregnancy without adverse obstetrical events in primary antiphospholipid syndrome: French phase II multicenter randomized trial, HYDROSAPL]. *Gynecol Obstet Fertil Senol.* (2018) 46:598–604. 10.1016/j.gofs.2018.06.008 30041771

[7] Albert CR Schlesinger WJ Viall CA Mulla MJ Brosens JJ Chamley LW et al. Effect of hydroxychloroquine on antiphospholipid antibody-induced changes in first trimester trophoblast function. *Am J Reprod Immunol*. (2014) 71:154–64. 10.1111/aji.12184 24325143

[8] De Carolis S Botta A Salvi S di Pasquo E Del Sordo G Garufi C et al. Is there any role for the hydroxychloroquine (HCQ) in refractory obstetrical antiphospholipid syndrome (APS) treatment? *Autoimmun Rev*. (2015) 14:760–2. 10.1016/j.autrev.2015.04.010 25936295

[9] Kravvariti E Koutsogianni A Samoli E Sfikakis PP Tektonidou MG. The effect of hydroxychloroquine on thrombosis prevention and antiphospholipid antibody levels in primary antiphospholipid syndrome: a pilot open label randomized prospective study. *Autoimmun Rev*. (2020) 19:102491. 10.1016/j.autrev.2020.102491 32084592

[10] Cervera R Khamashta MA Hughes GR. ‘Overlap’ syndromes. *Ann Rheum Dis*. (1990) 49:947–8. 10.1136/ard.49.11.947 2256747 PMC1004273

[11] Kelly A Panush RS. Diagnostic uncertainty and epistemologic humility. *Clin Rheumatol*. (2017) 36:1211–4. 10.1007/s10067-017-3631-8 28432522

[12] Singh M Wambua S Lee SI Okoth K Wang Z Fazla F et al. MuM-PreDiCT. Autoimmune diseases and adverse pregnancy outcomes: an umbrella review. *Lancet*. (2023) 402(Suppl 1):S84. 10.1016/S0140-6736(23)02128-1 37997130

[13] Zucchi D Tani C Mosca M. Reproductive Health in RA, Lupus, and APS. *J Clin Rheumatol.* (2024) 30(7S Suppl 1):S42–8. 10.1097/RHU.0000000000002141 39325124

[14] Miyakis S Lockshin MD Atsumi T Branch DW Brey RL Cervera R et al. International consensus statement on an update of the classification criteria for definite antiphospholipid syndrome (APS). *J Thromb Haemost*. (2006) 4:295–306. 10.1111/j.1538-7836.2006.01753.x 16420554

[15] Sciascia S Amigo MC Roccatello D Khamashta M. Diagnosing antiphospholipid syndrome: ‘extra-criteria’ manifestations and technical advances. *Nat Rev Rheumatol*. (2017) 13:548–60. 10.1038/nrrheum.2017.124 28769114

[16] Aringer M Costenbader K Daikh D Brinks R Mosca M Ramsey-Goldman R et al. 2019 European league against rheumatism/american college of rheumatology classification criteria for systemic lupus erythematosus. *Arthritis Rheumatol*. (2019) 71:1400–12. 10.1002/art.40930 31385462 PMC6827566

[17] Shiboski CH Shiboski SC Seror R Criswell LA Labetoulle M Lietman TM et al. International Sjögren’s Syndrome Criteria Working Group. 2016 American College of Rheumatology/European League Against Rheumatism classification criteria for primary Sjögren’s syndrome: a consensus and data-driven methodology involving three international patient cohorts. *Ann Rheum Dis*. (2017) 76:9–16. 10.1136/annrheumdis-2016-210571 27789466

[18] Ortega-Hernandez OD Shoenfeld Y. Mixed connective tissue disease: an overview of clinical manifestations, diagnosis and treatment. *Best Pract Res Clin Rheumatol*. (2012) 26:61–72. 10.1016/j.berh.2012.01.009 22424193

[19] Ferrazzani S Degennaro VA Di Stasio E Poppa G Moresi S Salvi S et al. Development of a new foetal growth curve from a large sample of Italian population. *Minerva Pediatr*. (2017) 69:245–50. 10.23736/S0026-4946.16.04258-4 26365747

[20] Hypertension in pregnancy. Report of the American College of Obstetricians and Gynecologists’ Task Force on Hypertension in Pregnancy. *Obstet Gynecol.* (2013) 122:1122–31. 10.1097/01.AOG.0000437382.03963.88 24150027

[21] World Health Organization [WHO]. *WHO Recommendations for Prevention and Treatment of Pre-Eclampsia and Eclampsia.* Geneva: WHO (2011). 23741776

[22] National Institute for Health and Care Excellence. *Hypertension in Pregnancy: Quality Standard.* Manchester: NICE (2013).

[23] Silver R Craigo S Porter F Osmundson SS Kuller JA Norton ME. Society for Maternal-Fetal Medicine Consult Series #64: systemic lupus erythematosus in pregnancy. *Am J Obstet Gynecol*. (2023) 228:B41–60. 10.1016/j.ajog.2022.09.001 36084704

[24] Tan Z Shao M Zhou Y Wang L Ma Y Xiang N et al. Increased risk of adverse gestational outcomes in pregnant women with primary Sjögren’s syndrome. *RMD Open.* (2024) 10:e003616. 10.1136/rmdopen-2023-003616 38806189 PMC11138269

[25] Chen J Huai J Yang H. Low-molecular-weight heparin for the prevention of preeclampsia in high-risk pregnancies without thrombophilia: a systematic review and meta-analysis. *BMC Pregnancy Childbirth.* (2024) 24:68. 10.1186/s12884-023-06218-9 38233773 PMC10792962

[26] Liu YH Zhang YS Chen JY Wang ZJ Liu YX Li JQ et al. Comparative effectiveness of prophylactic strategies for preeclampsia: a network meta-analysis of randomized controlled trials. *Am J Obstet Gynecol*. (2023) 228:535–46. 10.1016/j.ajog.2022.10.014 36283479

[27] Xu J Tang Y Peng B Zhang WH Wang X. Effect of low-molecular-weight heparin on placenta-mediated fetal growth restriction in a tertiary referral hospital: a 7-year retrospective cohort study. *Int J Gynaecol Obstet*. (2024) 165:220–8. 10.1002/ijgo.15098 37726961

[28] Guller S Kong L Wozniak R Lockwood CJ. Reduction of extracellular matrix protein expression in human amnion epithelial cells by glucocorticoids: a potential role in preterm rupture of the fetal membranes. *J Clin Endocrinol Metab*. (1995) 80:2244–50. 10.1210/jcem.80.7.7608287 7608287

[29] Lockwood CJ Radunovic N Nastic D Petkovic S Aigner S Berkowitz GS. Corticotropin-releasing hormone and related pituitary-adrenal axis hormones in fetal and maternal blood during the second half of pregnancy. *J Perinat Med*. (1996) 24:243–51. 10.1515/jpme.1996.24.3.243 8827573

[30] Østensen M Khamashta M Lockshin M Parke A Brucato A Carp H et al. Anti-inflammatory and immunosuppressive drugs and reproduction. *Arthritis Res Ther*. (2006) 8:209. 10.1186/ar1957 16712713 PMC1526635

[31] Shimada H Wakiya R Kanenishi K Miyatake N Nakashima S Mansour MMF et al. Preterm birth is strongly affected by the glucocorticoid dose during pregnancy in women complicated by systemic lupus erythematosus. *Arthritis Res Ther.* (2022) 24:10. 10.1186/s13075-021-02699-1 34980235 PMC8722014

[32] Bandoli G Palmsten K Forbess Smith CJ Chambers CD. A review of systemic corticosteroid use in pregnancy and the risk of select pregnancy and birth outcomes. *Rheum Dis Clin North Am*. (2017) 43:489–502. 10.1016/j.rdc.2017.04.013 28711148 PMC5604866

[33] Liu XX Zhu XM Miao Q Ye HY Zhang ZY Li YM. Hyperglycemia induced by glucocorticoids in nondiabetic patients: a meta-analysis. *Ann Nutr Metab*. (2014) 65:324–32. 10.1159/000365892 25402408

[34] Wei L MacDonald TM Walker BR. Taking glucocorticoids by prescription is associated with subsequent cardiovascular disease. *Ann Intern Med.* (2004) 141:764–70. 10.7326/0003-4819-141-10-200411160-00007 15545676

[35] Leong KH Koh ET Feng PH Boey ML. Lipid profiles in patients with systemic lupus erythematosus. *J Rheumatol.* (1994) 21:1264–7. 10.36347/sajb.2022.v10i11.010 7966068

[36] Kelly BA Lewandowski AJ Worton SA Davis EF Lazdam M Francis J et al. Antenatal glucocorticoid exposure and long-term alterations in aortic function and glucose metabolism. *Pediatrics*. (2012) 129:e1282–90. 10.1542/peds.2011-3175 22508917

[37] Dalziel SR Walker NK Parag V Mantell C Rea HH Rodgers A et al. Cardiovascular risk factors after antenatal exposure to betamethasone: 30-year follow-up of a randomised controlled trial. *Lancet.* (2005) 365:1856–62. 10.1016/S0140-6736(05)66617-2 15924982

[38] Ninan K Liyanage SK Murphy KE Asztalos EV McDonald SD. Evaluation of long-term outcomes associated with preterm exposure to antenatal corticosteroids: a systematic review and meta-analysis. *JAMA Pediatr.* (2022) 176:e220483. 10.1001/jamapediatrics.2022.0483 35404395 PMC9002717

[39] Costedoat-Chalumeau N Amoura Z Duhaut P Huong DL Sebbough D Wechsler B et al. Safety of hydroxychloroquine in pregnant patients with connective tissue diseases: a study of one hundred thirty-three cases compared with a control group. *Arthritis Rheum*. (2003) 48:3207–11. 10.1002/art.11304 14613284

[40] Sciascia S Hunt BJ Talavera-Garcia E Lliso G Khamashta MA Cuadrado MJ. The impact of hydroxychloroquine treatment on pregnancy outcome in women with antiphospholipid antibodies. *Am J Obstet Gynecol.* (2016) 214:273.e1–.e8. 10.1016/j.ajog.2015.09.078. 26429521

[41] Belizna C Pregnolato F Abad S Alijotas-Reig J Amital H Amoura Z et al. HIBISCUS: Hydroxychloroquine for the secondary prevention of thrombotic and obstetrical events in primary antiphospholipid syndrome. *Autoimmun Rev*. (2018) 17:1153–68. 10.1016/j.autrev.2018.05.012 30316994

[42] De Carolis S Tabacco S Rizzo F Giannini A Botta A Salvi S et al. Antiphospholipid syndrome: an update on risk factors for pregnancy outcome. *Autoimmun Rev*. (2018) 17:956–66. 10.1016/j.autrev.2018.03.018 30118899

[43] Hooper A Bacal V Bedaiwy MA. Does adding hydroxychloroquine to empiric treatment improve the live birth rate in refractory obstetrical antiphospholipid syndrome? A systematic review. *Am J Reprod Immunol*. (2023) 90:e13761. 10.1111/aji.13761 37641373

[44] Cai T Zhao J Yang Y Jiang Y Zhang JA. Hydroxychloroquine use reduces mortality risk in systemic lupus erythematosus: a systematic review and meta-analysis of cohort studies. *Lupus*. (2022) 31:1714–25. 10.1177/09612033221129774 36325952

[45] Clowse ME Magder L Witter F Petri M. Hydroxychloroquine in lupus pregnancy. *Arthritis Rheum*. (2006) 54:3640–7. 10.1002/art.22159 17075810

[46] Liu Y Zhang Y Wei Y Yang H. Effect of hydroxychloroquine on preeclampsia in lupus pregnancies: a propensity score-matched analysis and meta-analysis. *Arch Gynecol Obstet*. (2021) 303:435–41. 10.1007/s00404-020-05762-5 32880707

[47] Do SC Rizk NM Druzin ML Simard JF. Does hydroxychloroquine protect against preeclampsia and preterm delivery in systemic lupus erythematosus pregnancies? *Am J Perinatol*. (2020) 37:873–80. 10.1055/s-0039-3402752 31899930

[48] Hu Z Gao R Huang W Wang H Qin L. Effect of hydroxychloroquine on lupus activity, preeclampsia and intrauterine growth restriction in pregnant women with systemic lupus erythematosus and/or antiphospholipid syndrome: a systematic review and meta-analysis. *J Clin Med.* (2023) 12:485. 10.3390/jcm12020485 36675415 PMC9866542

[49] Barsalou J Costedoat-Chalumeau N Berhanu A Fors-Nieves C Shah U Brown P et al. Effect of in utero hydroxychloroquine exposure on the development of cutaneous neonatal lupus erythematosus. *Ann Rheum Dis*. (2018) 77:1742–9. 10.1136/annrheumdis-2018-213718 30297329 PMC6382275

[50] Izmirly PM Costedoat-Chalumeau N Pisoni CN Khamashta MA Kim MY Saxena A et al. Maternal use of hydroxychloroquine is associated with a reduced risk of recurrent anti-SSA/Ro-antibody-associated cardiac manifestations of neonatal lupus. *Circulation.* (2012) 126:76–82. 10.1161/CIRCULATIONAHA.111.089268 22626746 PMC3437628

[51] Pasquier E de Saint-Martin L Marhic G Chauleur C Bohec C Bretelle F et al. Hydroxychloroquine for prevention of recurrent miscarriage: study protocol for a multicentre randomised placebo-controlled trial BBQ study. *BMJ Open.* (2019) 9:e025649. 10.1136/bmjopen-2018-025649 30898821 PMC6527997

[52] Schreiber K Breen K Cohen H Jacobsen S Middeldorp S Pavord S et al. HYdroxychloroquine to improve pregnancy outcome in women with AnTIphospholipid Antibodies (HYPATIA) Protocol: a multinational randomized controlled trial of hydroxychloroquine versus placebo in addition to standard treatment in pregnant women with antiphospholipid syndrome or antibodies. *Semin Thromb Hemost*. (2017) 43:562–71. 10.1055/s-0037-1603359 28609801

[53] de Moreuil C Alavi Z Pasquier E. Hydroxychloroquine may be beneficial in preeclampsia and recurrent miscarriage. *Br J Clin Pharmacol*. (2020) 86:39–49. 10.1111/bcp.14131 31633823 PMC6983516

